# Simultaneous Visualization of Both Signaling Cascade Activity and End-Point Gene Expression in Single Cells

**DOI:** 10.1371/journal.pone.0020148

**Published:** 2011-05-25

**Authors:** Irene Weibrecht, Ida Grundberg, Mats Nilsson, Ola Söderberg

**Affiliations:** Department of Immunology, Genetics and Pathology, Science for Life Laboratory, Rudbeck Laboratory, Uppsala University, Uppsala, Sweden; Karolinska Institutet, Sweden

## Abstract

We have developed an approach for simultaneous detection of individual endogenous protein modifications and mRNA molecules in single cells *in situ*. For this purpose we combined two methods previously developed in our lab: *in situ* proximity ligation assay for the detection of individual protein interactions and -modifications and *in situ* detection of single mRNA molecules using padlock probes. As proof-of-principle, we demonstrated the utility of the method for simultaneous detection of phosphorylated PDGFRβ and *DUSP6/MKP-3* mRNA molecules in individual human fibroblasts upon PDGF-BB stimulation. Further we applied drugs disrupting the PDGFRβ signaling pathway at various sites to show that this combined method can concurrently monitor the molecular effect of the drugs, i.e. inhibition of downstream signaling from the targeted node in the signaling pathway. Due to its ability to detect different types of molecules in single cells *in situ* the method presented here can contribute to a deeper understanding of cell-to-cell variations and can be applied to e.g. pinpoint effector sites of drugs in a signaling pathway.

## Introduction

Studying molecules in their natural cellular context provides valuable means to learn more about the complex function and regulation of cells. Cellular activity can be determined at several levels of complexity. Protein or mRNA expression can be used to determine endpoint effects of an active signaling pathway, but in order to investigate propagation of the signals assays for protein interactions and post-translational modifications are needed. So far most of the studies on protein-protein interactions and -modifications as well as mRNA-expression levels have been performed in a bulk population, rather than in individual cells, due to restrictions in detection sensitivity and specificity of the methods available. These methods yield average information about the molecule of interest but will fail to detect intercellular variation [1]. Studies that are based on single cell data circumvent the risk that rare events are hidden within the bulk population or that the average for the whole population is not reflecting real variations within it. This is of particular importance when studying cellular signaling, since each cell in a population – even if they are clonal expansions from one precursor – will answer slightly different to a stimulus due to variations in e.g. cell cycle, accessibility to ligand and transcription factors, accumulated mutations and the cocktail of other signals this cell is receiving [2], [3]. Visualization of multiple nodes in a signaling pathway will enhance the ability to monitor signal progression and provide a better tool to address heterogeneity in the response of a cell population to stimulation, enabling studies on cellular communication.

**Figure 1:**
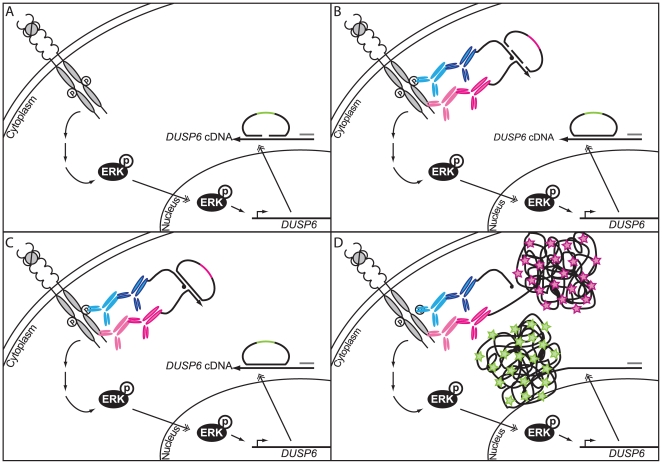
Method overview. (A) Upon stimulation with PDGF-BB the PDGFRβ becomes autophosphorylated at various sites. This promotes downstream signaling to ERK activation. Phosphorylated ERK translocates into the nucleus and enhances *DUSP6* expression. Upon fixation of the cells *DUSP6* mRNA is reverse transcribed utilizing an LNA-modified primer. Subsequently the cDNA is made accessible through RNase H digestion. The single standed cDNA stays attached to the mRNA-primer by the 5′-end of the primer that contains the LNA bases, which is not recognized by RNase H – to allow hybridization of padlock probes. (B) After ligation of the padlock probe to a circular DNA molecule, primary antibodies directed against the PDGFRβ and phosphorylated tyrosines, followed by addition of secondary PLA probes, are applied for detection of the phosphorylated PDGFRβ. If the antibodies and probes are bound in close proximity two circularization oligonucleotides can hybridize to the PLA probes. (C) This molecule can subsequently also be joined by ligation, giving rise to another circular DNA molecule. (D) Both DNA circles are then simultaneously amplified by RCA, primed from either the cDNA or the oligonucleotide attached to the PLA probe. The resulting bundles of DNA (amplified ∼1000 times) are still attached to the place where the RCA was primed and can be detected by hybridization of fluorescence labeled oligonucleotides (different fluorophores (green and pink stars) for the two types of circles), resulting in bright spots easily distinguishable from background.

To facilitate investigations of endogenous protein interactions and post-translational modifications *in situ* we recently developed the *in situ* proximity ligation assay (*in situ* PLA [4], [5]). For detection of DNA and individual mRNA molecules *in situ* we developed the padlock probes that enable discrimination between single nucleotide polymorphisms (SNP:s) [6], [7]. Both methods allow the detection of single molecules – either proteins or nucleic acid molecules – in fixed cells and tissues. Briefly, *in situ* PLA as applied here utilizes two antibodies from different species, one directed against the protein of interest, the other against its posttranslational modification. Upon binding to the same modified protein, species-specific PLA probes directed against the primary antibodies and carrying two different oligonucleotides are added (Figure 1). Only if these bind in close proximity to the same target protein will they guide the hybridization and ligation of two connector oligonucleotides, generating a circular DNA molecule that is amplified by a strictly circle-dependent rolling circle amplification (RCA) using phi29 DNA polymerase. This reaction is primed from one of the oligonucleotides attached to the PLA probes and thus the single stranded RCA product (RCP) will stay attached to the place where the PLA probe was bound. The RCP collapses into a bundle of DNA, consisting of ∼1000 repetitive elements, complementary to the DNA circle. This bundle can then be easily detected by hybridization with fluorescence labeled detection oligonucleotides, concentrating ∼1000 fluorophores in a sub-µm sized bright spot, to be visualized by fluorescence microscopy.

Padlock probes for detection of individual mRNA molecules [7] also utilize RCA to visualize the detected molecules. The mRNA molecules are first reverse transcribed into cDNA, using a specific LNA-primer, to enable ligation of the padlock probes on cDNA target molecules, which is a more efficient target than RNA. To create a single-stranded cDNA target, the original mRNA is digested by RNase H, except for the part that is tightly hybridized to the LNA-modified nucleotides of the primer. This will keep the synthesized cDNA anchored to the mRNA in the cell. A padlock probe is then hybridized to the cDNA and upon perfect match the target complementary 5′ and 3′ ends will become joined by ligation, creating a circular DNA molecule (Figure 1). Since the ligase is very sensitive to mismatches at the ligation junction, padlock probes can be used for discrimination of SNP:s *in situ* [6], [7], [8]. After circularization, the padlock probe is used as template for an RCA primed from the cDNA, ensuring that the RCP stays attached to the original target position in the cell. Visualization is subsequently done as described for *in situ* PLA. As both methods outlined here are able to investigate individual molecules of different types we wanted to combine *in situ* PLA and padlock probes for detection of individual protein modifications and mRNA molecules to provide an assay to measure activity at different positions in the signaling pathway, giving a more coherent view on the status of individual cells.

The platelet-derived growth factor receptor beta (PDGFRβ) is a receptor tyrosine kinase associated with growth and motility. Upon stimulation of PDGFRβ with PDGF-BB the receptor is dimerized and auto-phosphorylated at several sites leading to recruitment of GRB2 and SOS, which in turn activate the RAS-RAF-MEK-ERK pathway. Upon sustained stimulation with PDGF-BB the phosphorylated receptor becomes internalized while activated ERK upregulates expression of downstream mRNA-targets e.g. dual specificity phosphatase 6 (*DUSP6*), also called *MKP-3* [9], [10], a dual-specificity phosphatase that dephosphorylates ERK, thereby providing a negative feedback loop for its own expression [10].

We used this model system to visualize the kinetics of ligand stimulation at an initial stage – phosphorylation of the PDGFRβ – and a late stage – expression of the downstream target gene *DUSP6*. Further, we applied drugs that target different nodes in the pathway to test if the assay will be applicable to pinpoint the molecular effect of a drug. Using the assay developed above, treatment with drugs caused inhibition of either both phosphorylation of the PDGFRβ and *DUSP6* expression or *DUSP6* expression alone, depending on where on the pathway they act. We demonstrated detection of cell-to-cell variations with single molecule resolution in fixed cells. The method presented here could be especially valuable when screening for new drugs, where the effector molecules are still unknown or where the drugs should only target a certain part of a signaling pathway.

## Materials and Methods

### Cell culture

TERT immortalized human fibroblast (BJhTert) [11] cells were seeded on 8-well chamber slides (LabTek, Nunc), 20,000 cells per well. The cells were allowed to attach overnight and subsequently starved in Modified Eagle Medium (MEM, Gibco)+0.1% FCS (heat-inactivated, Sigma) for 48 h. For stimulation PDGF-BB was added to a final concentration of 100 ng ml^−1^ and the cells were incubated at 37°C for the indicated time points. If the cells were treated with drugs, the drugs Gleevec (Novartis) and 5-Iodotubercidin (Sigma), dissolved in DMSO (Sigma) to a 10 mM stock concentration, were applied at a final concentration of 10 µM in fresh MEM+0.1% FCS for 1 h at 37°C before PDGF-BB was added at the indicated time points. The drug concentration was held constant throughout the experiment.

For fixation the cells were put on ice and the medium was immediately exchanged for ice-cold DEPC (Applichem Lifescience)-treated PBS (DEPC-PBS, 900 µl per well). After a 1 min wash, the cells were fixed in 2% (w/v) paraformaldehyde (PFA, Sigma Aldrich) in DEPC-PBS for 30 min at room temperature (20–23°C). Prior to permeabilization in ice cold 70% ethanol for 30 min on ice, the cells were washed again in ice-cold DEPC-PBS. Now the silicon mask of the chamber slides was removed, the slides were dehydrated through a series of 70%, 85% and 99.5% ethanol for ∼1 min each, hydrophobic barrier pen was applied at the borders of the slide wells and finally 8-chamber Secure Seals (9 mm in diameter, 0.8 mm deep; Grace Bio-Labs) were attached to the slides.

### Preparation of secondary PLA probes

One mg donkey anti-rabbit IgG (711-005-152, Jackson Immunoresearch) and 1 mg donkey anti-mouse IgG (715-005-150, Jackson Immunoresearch) were concentrated over Amicon ultra 0.5 ml, 10 kDa (UFC501008, Millipore) and dialyzed in 7 kD dialysis cup (Slide-A-Lyzer MINI dialysis units, #69562, Pierce) against PBS over night at 4°C. Antibodies were activated in SMCC (Pierce) added in 25-fold excess over antibody for 2 h at room temperature. Primer oligonucleotide (5′ thiol-AAA AAA AAA ATA TGA CAG AAC TAG ACA CTC TT (Eurogentec)) and blocked oligonucleotide (5′ thiol-AAA AAA AAA AGA CGC TAA TAG TTA AGA CGC TTU UU (Biomers)) were degassed at 95°C for 3 min and reduced by incubation with 25 mM DTT for 1 h at 37°C. Both, antibodies and oligonucleotides were purified using an Illustra NAP-5 column (#17-0853-01, GE Healthcare), equilibrated and eluted with PBS+5 mM EDTA pH 7.5. Afterwards anti-rabbit IgG was immediately mixed with the primer oligonucleotide and anti-mouse IgG with the blocked oligonucleotide. PLA probes were then dialyzed against PBS at 4°C overnight. Before purification by HPLC the conjugates were concentrated to 25 µl by Amicon ultra 0.5 ml, 10 kDa columns and 10 mM DTT was added to the conjugate to reduce unbound oligonucleotide dimers. The conjugates were incubated for 10 min at room temperature and subsequently injected into HPLC (Hitachi D7000 series). Chromatography was done over a Superdex 75 PC 3.2/30 column with a flow rate of 0.06 ml min^−1^ to separate conjugates from free oligonucleotides and samples were collected at 16–20.5 min (conjugate peak).

### Detection of phosphorylated PDGFRβ with *in situ* PLA

The protocol is based on the protocol described previously in Jarvius *et al.* 2007 [5]. All reactions were performed in Secure Seals attached to the slides. Prior to each new step all liquids were removed from the Secure Seal chambers and for incubations exceeding 30 min the chambers were sealed with q-PCR film to avoid evaporation. All washes were performed by flushing the wells with ∼500 µl of the appropriate washing buffer.

First the wells were rehydrated in 1× PBS-T (DEPC-PBS+0.05% Tween20 (Sigma)) for 10 min at room temperature. The cells were blocked in Protein-Block, serum free (Dako), 2.5 ng µl^−1^ sonicated salmon sperm DNA (Invitrogen) and 2.5 mM L-cysteine (Sigma) for 90 min at 37°C. After blocking primary antibodies (63 ng ml^−1^ rabbit-anti-PDGFRβ (#3169 Cell Signaling) and 6 µg ml^−1^ mouse-anti-pY100 (#9411, Cell signaling)) were applied in blocking solution over night at 4°C. Primary antibodies were removed by flushing the chambers with ∼500 µl 1× PBS-T. Secondary PLA probes (1.6 ng µl^−1^ donkey-anti-rabbit-primer and 5 ng µl^−1^ donkey-anti-mouse-blocked, final concentration, see above) were incubated separately in half the final volume of blocking solution for 30 min at room temperature before they were mixed and applied to the cells for 1 h at 37°C. Subsequently the cells were washed in 10 mM Tris HCl pH 7.5, 0.1% Tween20 for 5 min at 37°C and once with 1× PBS-T. Hybridization was done with 125 nM connector oligonucleotides (5′ phosphate-CTA TTA GCG TCC AGT GAA TGC GAG TCC GTC TAA GAG AGT AGT ACA GCA GCC GTC AAG AGT GTC TA (Eurogentec) and 5′-phosphate-GTT CTG TCA TAT TTA AGC GTC TTA A (Eurogentec)) in 1× ligation buffer (10 mM Tris acetate, pH 7.5, 10 mM magnesium acetate, 50 mM potassium acetate), 0.25 µg µl^−1^ BSA (New England Biolabs), 0.05% Tween20, 250 mM NaCl for 30 min at 37°C. After a wash in 1× PBS-T ligation was performed in 1× ligation buffer, 0.05 U µl^−1^ T4-DNA ligase (Fermentas), 1 mM ATP (Fermentas), 0.25 µg µl^−1^ BSA, 0.05% Tween20 and 250 mM NaCl for 30 min at 37°C. Subsequent to an additional washing step in 1× PBS-T, rolling circle amplification was done with 0.25 U µl^−1^ phi29 DNA polymerase (Fermentas) in 1× RCA buffer (50 mM Tris-HCl, 10 mM MgCl_2_, 10 mM (NH_4_)_2_SO_4_, pH 7.5), 0.25 mM dNTP (Fermentas), 0.2 µg µl^−1^ BSA, 5% glycerol (Sigma) 90 min at 37°C. Before detection with 100 nM detection oligonucleotide (5′ Alexa 555-CAG TGA ATG CGA GTC CGT CT 3′ (Trilink)) in 2× SSC, 0.25 µg µl^−1^ BSA, 75 ng µl^−1^ poly(A) (Sigma) and 0.05% Tween20, the cells were washed once in 1× PBS-T. After detection Secure Seals were removed and the slides were washed twice in TBS for 10 min each. The cytoplasm of the cells was thereafter counterstained with 2.5 µg ml^−1^ WGA 488 (Invitrogen) diluted in 1× DEPC-PBS for 30 min at room temperature. Final washes were done 2× 10 min in TBS before the slides were spun dry and mounted in Vectashield mounting medium (Vector)+100 ng ml^−1^ DAPI.

### *In situ* detection of *DUSP6* transcripts using padlock probes and target-primed RCA

Primers and padlock probes were designed for the *DUSP6* and *ACTB* transcripts using GenBank accession numbers NM_001946 (*DUSP6*) and NM_001101.3 (*ACTB*). The primers used for *in situ* synthesis were for *DUSP6* 5′-C**C**G **C**T**G** G**C**T **C**T**T** A**G**T GTC AAT GAA T-3′ (Exiqon) and for *ACTB* transcripts 5′-G**T**G **G**A**C** G**G**G **C**G**G** C**G**G ATC GGC AAA G-3′ (Exiqon). Bold letters represent LNA-modified bases. The padlock probes and detection oligonucleotides applied for detection of *DUSP6* and *ACTB* transcripts were 5′ phosphate- GAG AGA GAT TCA TTG TTC CTT TTA CGA *CCT CAA TGC TGC TGC TGT ACT AC*T CTT CCT GGG CAG CTT CAT T (Integrated DNA Technology) for *DUSP6* and 5′ phosphate- AGC CTC GCC TTT GCC TTC CTT TTA CGA *CCT CAA TGC ACA TGT TTG GCT CC*T CTT CGC CCC GCG AGC ACA G (Integrated DNA Technology) for *ACTB*. Underscored letters denotes the target complementary ends and italic letters represent the site and the sequence of the respective detection oligonucleotides. The detected *DUSP6*-transcripts were Cy3-labeled (MWG) for the individual time series experiments and Cy5-labeled (Eurogentec) in all other experiment. *ACTB*-transcripts were Cy5-labeled (Integrated DNA Technology).

The *in situ* detection-procedure was performed in a similar way as described by Larsson et al. [7]. Fixation, permeabilization and dehydration of cells were however done as described above. Subsequent to the application of the Secure Seals, the cells were rehydrated in PBS-T. Reverse transcription was performed with 1 µM of the appropriate LNA-primer in 20 U µl^−1^ RevertAid H minus M-MuLV reverse transcriptase (Fermentas), 0.5 mM dNTPs, 0.2 µg µl^−1^ BSA, 1 U µl^−1^ Ribolock RNase inhibitor (Fermentas) in M-MuLV reaction buffer. Slides were incubated for 3 h at 37°C and then washed twice in PBS-T. All subsequent washes were performed in the same way. Postfixation was done in 2% (w/v) paraformaldehyde for 30 min at room temperature before the slides were washed. To make the cDNA accessible for padlock probe hybridization and to simultaneously ligate the padlock probe to its target sequence, the cells were incubated with 0.1 µM padlock probe, 0.4 U µl^−1^ RNase H (Fermentas), 0.5 U µl^−1^ Ampligase (Epicentre), 1 U µl^−1^ Ribolock RNase inhibitor in Ampligase buffer (20 mM Tris-HCl, pH 8.3, 75 mM KCl, 10 mM MgCl_2_, 0.5 mM NAD and 0.01% Triton X-100), 20% formamide (Merck), first for 30 min at 37°C and then for 45 min at 45°C. After an additional wash RCA was performed with 1 U µl^−1^ phi29 DNA polymerase, 1 U µl^−1^ Ribolock RNase inhibitor in the supplied phi29 DNA polymerase reaction buffer, 0.25 mM dNTPs, 0.2 µg µl^−1^ BSA and 5% glycerol for 90 min at 37°C. RCPs were labeled with 0.25 µM detection oligonucleotide in 2× SSC and 20% formamide for 15 min at 37°C. After detection the slides were washed twice in PBS-T and Secure Seals were removed. The cytoplasm of the cells was thereafter counterstained and the slides mounted as described above.

### Simultaneous *in situ* detection of individual phosphorylated PDGFRβ and *DUSP6* or *ACTB* mRNA

The procedure for simultaneous detection of individual proteins and transcripts in single cells was based on merging the two protocols described above. The merged protocol started with *in situ* reverse transcription, converting mRNA to *DUSP6* and *ACTB* cDNA. This was followed by post-fixation of the cells, digestion by RNase H and hybridization/ligation of the specific padlock probes to the target cDNA molecules. These steps were performed in the same fashion as described above for solely transcript detection. Thus, the padlock probes were locked onto their targets and the target transcripts were secured. The combined protein/mRNA protocol could subsequently proceed with the detection of phosphorylated PDGFRβ as described above except that 1 U µl^−1^ Ribolock RNase inhibitor was added to the blocking, antibody and PLA probe mixtures and DEPC-ddH_2_O was used instead of ddH_2_O in hybridization and ligation. RCA was performed as described for *in situ* PLA except that 2.5 U µl^−1^ phi29 DNA polymerase were used and both, padlock probes and *in situ* PLA signals were amplified. For detection, the fluorescence labeled oligonucleotides (see above), with a final concentration of 100 nM, were added to the detection mix described for *in situ* PLA.

### Image acquisition

Images were acquired using a Zeiss Axioplan 2 epifluorescence microscope, the AxioCam MRm CCD sensor and a 20×/0.8 Plan Apochromat lens together with filters for DAPI, FITC, Cy3 and Cy5. For each condition z-stacks were acquired at three positions of the well and the positions were chosen using the FITC channel (cytoplasm staining).

### Image analysis and illustrations

Image J 1.44 d was used to make maximum intensity projections (MIPs) of the image stack of each channel. In the FITC channel, ImageJ was applied to enhance the contrast, subtract background (rolling ball radius = 200 pixel) and run a median filter (radius 4 pixel). Background was also subtracted from DAPI-images (rolling ball radius = 50 pixel).

Subsequently, CellProfiler v.10415 was used to identify cell nuclei (thresholding method: two-class thresholding “Otsu PerObject”) in previously background subtracted DAPI images. The nuclei were then combined with background corrected FITC images to identify the cytoplasm of individual cells (method to identify objects: “Watershed - Image”, thresholding method: two-class thresholding “Otsu Adaptive”). Signals in Cy3 (PDGFRβ) and Cy5 (*DUSP6* or *ACTB*) were enhanced (feature type: “speckles”, feature size: 10), counted (diameter: 2–20 pixel, thresholding method: “RobustBackground Global” (for Cy3), diameter: 1–20 pixel, thresholding method “RobustBackground Adaptive” (for Cy5), threshold correction factor: 0.8) and related to the individual cells. Subsequently, if images did not contain signals, the amount of signals had to be manually corrected, since then the adaptive enhancement of Cy3 and Cy5 signals gave rise to false positive signals. Graphs were done in R [12]. In Figure 1B images were for better visualization treated in ImageJ as described here: One slice of the DAPI z-stack was selected, background was subtracted in ImageJ and thresholded for brightness and contrast. Cy3 and Cy5 z-stacks were projected to MIPs, the background was removed, brightness and contrast adjusted and a maximum filter (size: 1 pixel) was applied.

## Results

### Kinetics of PDGFRβ phosphorylation and *DUSP6* expression

**Figure 2:**
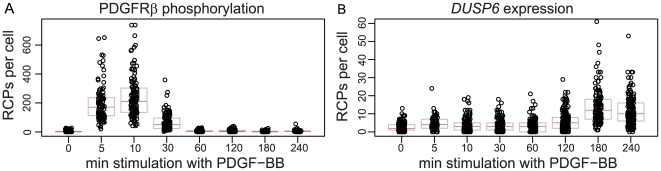
Individual detection of single phosphorylated PDGFRβ and *DUSP6* molecules in BJhTert cells. (A) Detection of individual phosphorylated PDGFRβ using *in situ* PLA in PDGF-BB-stimulated cells. Black circles represent the numbers of RCPs detected in individual cells (in total ∼90–130 cells per condition), the black bar represents the median of the population and grey boxes the 25% and 75% quartiles. The experiment is a representative example of three replicate experiments. (B) Detection of individual *DUSP6* mRNA molecules using padlock probes in BJhTert cells stimulated with PDGF-BB for different length of time. Black circles represent the numbers of RCPs detected in individual cells (in total ∼70–180 cells per condition), the black bar represents the median of the population and grey boxes the 25% and 75% quartiles. The experiment is a representative example of three replicate experiments.

To determine the kinetics of PDGFRβ phosphorylation and *DUSP6* expression upon stimulation with PDGF-BB, BJhTert cells were starved for 48 h prior to treatment with 100 ng ml^−1^ PDGF-BB. The cells were stimulated for different length of time (0 min–240 min) and subsequently fixed. Thereafter the phosphorylation of PDGFRβ was investigated using *in situ* PLA with one antibody directed against the receptor and one against phosphorylated tyrosine residues. In parallel, the expression on *DUSP6* transcripts was monitored by padlock probes on separate slides (Figure 2). As expected, the phosphorylation of PDGFRβ first increased substantially during the initial time points, peaking at 5–10 min after stimulation, before it gradually decreased again towards background levels due to internalization of the receptor [9]. In accordance with what has been reported before [10], *DUSP6* transcript levels remained constant for the first 30 min of stimulation, and then showed an increase until 3 h. Interestingly, the distribution in numbers of detected molecules per cell in the cell population is quite large, especially for PDGFRβ phosphorylation in response to stimulation. Regarding *DUSP6* expression, not all cells are responding to stimulation with PDGF-BB to the same extent. It rather appears that, for the time investigated, some cells burst with transcription while in others the signal is not propagated to the induction of *DUSP6* expression.

### Combined detection of protein modification and mRNA expression

**Figure 3:**
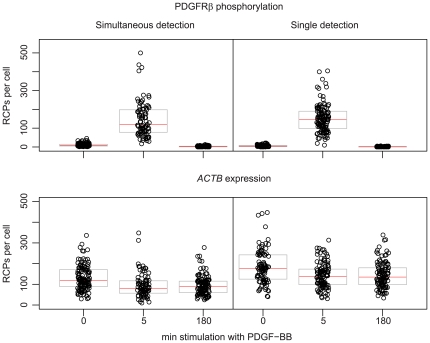
Individual and simultaneous detection of phosphorylated PDGFRβ and *ACTB* mRNA molecules in PDGF-BB stimulated BJhTert cells. Black circles represent the numbers of RCPs detected per cell (in total ∼85–130 cells per condition), black bars represent the median of the population and grey boxes the 25% and 75% quartiles. The experiment is a representative example of three replicate experiments. The result is presented in separate figures for PDGFRβ phosphorylation and *ACTB* expression, although recorded together.

Next, we tested the combined detection of protein modifications and single transcripts (Figure 1). For this purpose phosphorylation of PDGFRβ and *ACTB* mRNA molecules – an abundantly expressed housekeeping gene to enable a more detailed analysis of detection efficiency – were detected either individually or simultaneously. The cells were fixed and permeabilized before cDNA synthesis and padlock probe hybridization and ligation were performed. After the *ACTB* cDNA had been targeted, the cells were blocked and stained with *in situ* PLA reagents required to detect phosphorylated PDGFRβ. Both, reacted padlock probe and *in situ* PLA probes were amplified in the same reaction by RCA and detected by hybridization of fluorescence labeled detection oligonucleotides. We investigated whether the combined detection shows similar results as when the two detection reactions were run separately, and whether there is a decrease in detection efficiency upon combining the two. BJhTert cells were stimulated with PDGF-BB for 0 min, 5 min or 180 min. The results of the combined detection were consistent with the results of individual staining and the levels of *ACTB* transcripts were unaffected by stimulation with PDGF-BB, as expected. The combined protocol leads to a decrease in detected signals for both *in situ* PLA and padlock probes of about 50% compared to when performed in separate reactions (Figure 3).

### Drug induced inhibition of PDGFRβ phosphorylation and *DUSP6* expression

**Figure 4:**
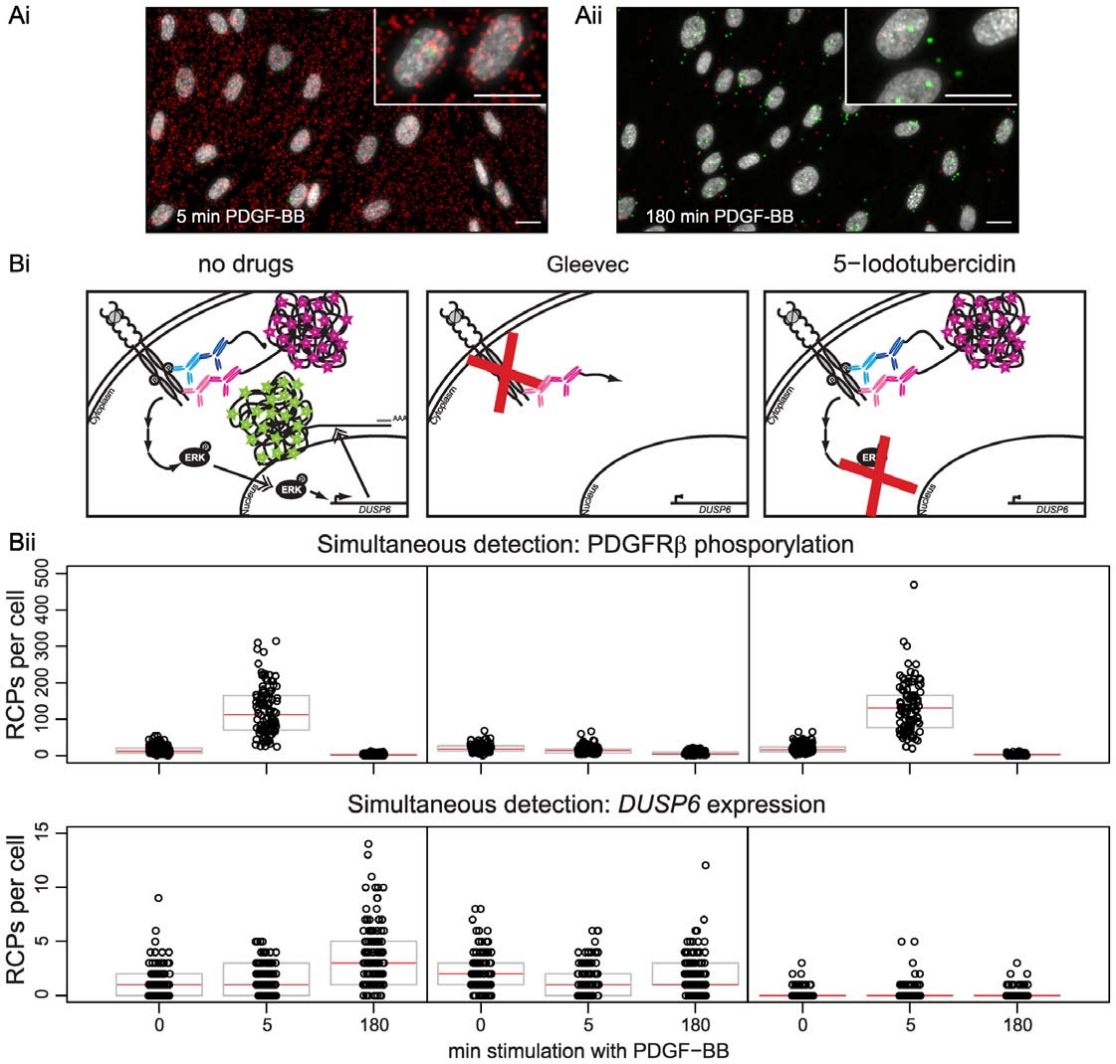
Simultaneous detection of individual phosphorylated PDGFRβ and *DUSP6* mRNA molecules in BJhTert cells. Cells were stimulated for (Ai) 5 min or (Aii) 180 min with PDGF-BB. *In situ* PLA signals, derived from PDGFRβ phosphorylation, are shown as red dots while *DUSP6* molecules are shown as green dots. The nuclei are shown in grey. Inserts represent magnified views over random cells. Scalebars represent 20 µm. (B) Simultaneous detection of individual phosphorylated PDGFRβ and *DUSP6* mRNA molecules cells treated with Gleevec or 5-Iodotubercidin at different time points after PDGF-BB stimulation. (Bi) Schematic illustration of the different drug target sites in PDGFRβ pathway and the consequences for signaling and expected RCPs. (Bii) Simultaneous detection of individual phosphorylated PDGFRβ and *DUSP6* mRNA molecules in BJhTert cells treated with Gleevec or 5-Iodotubercidin, at different time points after PDGF-BB stimulation. Black circles represent the numbers of RCPs detected per cell (in total ∼100–140 cells per condition), the red bar represents the median of the population and grey boxes the 25% and 75% quartiles. The experiment is a representative example of three replicate experiments. The result is presented in separate figures for PDGFRβ phosphorylation and *DUSP6* expression, although recorded together.

Having developed a method that enabled measurement of both protein activity and mRNA expression in single cells, we then tested if the assay would be suitable to detect perturbation of the PDGFRβ-signaling pathway using drugs that affect the pathway at different levels. In that way we were able to investigate if the assay could help in zooming in on the action of a drug. As proof of principle, we examined the effect of Gleevec (a tyrosine kinase inhibitor [13] targeting the PDGFRβ directly) and 5-Iodotubercidin (an inhibitor of ERK2 activation [14], located downstream of PDGFRβ but upstream of the induction of *DUSP6* transcription) on phosphorylation of PDGFRβ and *DUSP6* expression. As expected, treatment with Gleevec eliminated the increase in signals for both molecules, since it inhibits the activation of the PDGFRβ and thus all downstream events. 5-Iodotubercidin, on the other hand, did only eliminate the upregulation of *DUSP6* but not phosphorylation of the PDGFRβ, since it affected the signaling downstream of the receptor but upstream of *DUSP6* (Figure 4).

## Discussion

We herein successfully combined *in situ* PLA and padlock probes for detection of individual phosphorylated PDGFRβ and *DUSP6* transcripts in a single cell assay. First, the kinetics of each molecule was investigated in separate reactions and both molecules were detected as expected. The level of *DUSP6* expression previously reported, ∼1% compared to *ACTB* [15], are in agreement with the levels determined herein, confirming the ∼30% efficiency of mRNA detection using padlock probes *in situ* [7]. For protein detection the efficiency of *in situ* PLA is less, a few percent, depending on antibody affinity and quality of the PLA probes. However, detection of phosphorylated PDGFRβ using *in situ* PLA correlates with levels determined in a population based assay using immunoblot [5]. Combining *in situ* PLA with padlock probes detecting mRNA *in situ* did not affect detection of phosphorylated PDGFRβ upon stimulation with PDGF-BB. *ACTB* mRNA levels were largely unaffected by stimulation with PDGF-BB (Figure 3). However, the combined detection scheme decreased the signals for both targets to ∼50% compared to individual detection, most likely since the protocol is longer and involves more washing steps than individual detection.

We then demonstrated in a proof-of-principle experiment how this combined detection could be applied for screening of new drugs or signal transduction studies, treating BJhTert cells with either Gleevec or 5-Iodotubercidin. Subsequently PDGF-BB was added at different time points and phosphorylation of PDGFRβ, as well as expression of *DUSP6* transcripts, was measured in the same cells. As expected, treatment with Gleevec inhibited PDGFRβ phosphorylation, and consequently also impaired the induction of *DUSP6* expression. 5-Iodotubercidin inhibited ERK2 activation and therefore did not affect PDGFRβ phosphorylation but did inhibit *DUSP6* expression, which is dependent on ERK2 activation.

Both *in situ* PLA and padlock probes allow detection of individual target molecules at endogenous levels in individual cells. Even after synchronization of the cells by serum starvation for 48 h prior to stimulation, there was still a broad distribution in how many target molecules per cell that were detected. The difference in amount of RCPs per cell can partially be explained by differences in cell size but might also reflect differences in response time or intensity. Especially when looking at *DUSP6* transcript expression, some cells seem to burst with expression, while others remain at background levels, as previously described for other mRNA molecules [3]. As the measurements are performed in fixed samples, transient interactions/activations and bursts of expression between the different time points will not be recorded. Nevertheless, the assay provides information on frequency of expressing cells and levels of expression at the time of analyses. This cell-to-cell heterogeneity would have gone undetected if instead an averaging method would have been used. The possibility to simultaneously measure multiple parameters provides an advantage when more complex cell populations are studied, e.g. tissue sections, as they consists of a mixture of cell types, at various differentiation stages, responding to various external stimuli. Moreover, a simultaneous detection is advantageous in cases with rare or/and small sample size.

The combined method for detection of individual protein modifications and individual mRNA molecules in single cells *in situ* presented here may be a valuable tool to enable broader studies of signaling pathways. A further advantage: as the method is based upon imaging, differences between individual cells with respect to target molecule location, amount and modifications can be investigated together with morphological characteristics of the cells. Importantly, data about protein modification and mRNA expression can be related to the same cell. This might be of particular interest when new drugs are tested, especially such that should act very specifically and locally in a signaling pathway. In this paper we show detection of one specific post-translational modification and one specific transcript, but further developments of each individual method could also be applied to this combined technique. The main advantage with the strategy described herein lies in simultaneous targeting of multiple nodes in a signaling cascade to determine how a drug effects the propagation of the signals. This will allow studies in heterogeneous populations and provides a tool to determine if a drug can rewire a signaling circuit i.e., changing the balance in nodes where there are alternative interaction partners. In that way more branch points and levels of regulation can be revealed simultaneously, providing additional information on the activity status in individual cells that will reduce cost and increase throughput in high content analyses.
